# Supplementary material for: From sequence to enzyme mechanism using multi-label machine learning
Source: BMC Bioinformatics. 2014 May 19;15:150. doi: 10.1186/1471-2105-15-150 (PMC4229970; doi:10.1186/1471-2105-15-150)
Supplement: Additional file 2 — Java code of ml2db. Additional file ml2db_code.tar.gz contains the Java source code to run the multi-label machine learning experiments and save the results to database. The code’s Javadoc is included. [file 1471-2105-15-150-S2.zip › additional file 2/ml2db/ml2db/doc/main/ml2db/al/utility/neighbours/LinearMultiLabelNNSearch.html]

LinearMultiLabelNNSearch


JavaScript is disabled on your browser.


- Overview
- Package
- Class
- Use
- Tree
- Deprecated
- Index
- Help

- Prev Class
- Next Class

- Frames
- No Frames

- All Classes

- Summary:
- Nested |
- Field |
- Constr |
- Method

- Detail:
- Field |
- Constr |
- Method


main.ml2db.al.utility.neighbours

## Class LinearMultiLabelNNSearch

- java.lang.Object
- - weka.core.neighboursearch.NearestNeighbourSearch
  - - weka.core.neighboursearch.LinearNNSearch
    - - main.ml2db.al.utility.neighbours.LinearMultiLabelNNSearch

- All Implemented Interfaces:
  :   java.io.Serializable, weka.core.AdditionalMeasureProducer, weka.core.OptionHandler, weka.core.RevisionHandler

  Direct Known Subclasses:
  :   LinearNNSearchSkipInstanceItself

  ---

    

  ```
  public class LinearMultiLabelNNSearch
  extends weka.core.neighboursearch.LinearNNSearch
  ```

  Class

  Version:
  :   22 Jul 2011

  Author:
  :   Luna De Ferrari ldf(at)st-andrews.ac.uk

  See Also:
  :   Serialized Form

- - ### Constructor Summary

    Constructors

    Constructor and Description

    `LinearMultiLabelNNSearch(IdMultiLabelInstances instances)`
  - ### Method Summary

    Methods

    | Modifier and Type | Method and Description |
    | --- | --- |
    | `int[]` | `getLabelIndices()` |
    | `int` | `getNumberOfLabels()` |
    | `void` | `rebuild(IdMultiLabelInstances instances)` |

    - ### Methods inherited from class weka.core.neighboursearch.LinearNNSearch

      `addInstanceInfo, getDistances, getOptions, getRevision, getSkipIdentical, globalInfo, kNearestNeighbours, listOptions, nearestNeighbour, setInstances, setOptions, setSkipIdentical, skipIdenticalTipText, update`
    - ### Methods inherited from class weka.core.neighboursearch.NearestNeighbourSearch

      `combSort11, distanceFunctionTipText, enumerateMeasures, getDistanceFunction, getInstances, getMeasure, getMeasurePerformance, getPerformanceStats, measurePerformanceTipText, quickSort, setDistanceFunction, setMeasurePerformance`
    - ### Methods inherited from class java.lang.Object

      `equals, getClass, hashCode, notify, notifyAll, toString, wait, wait, wait`

- - ### Constructor Detail


    - #### LinearMultiLabelNNSearch

      ```
      public LinearMultiLabelNNSearch(IdMultiLabelInstances instances)
      ```
  - ### Method Detail


    - #### getLabelIndices

      ```
      public int[] getLabelIndices()
      ```


    - #### getNumberOfLabels

      ```
      public int getNumberOfLabels()
      ```


    - #### rebuild

      ```
      public void rebuild(IdMultiLabelInstances instances)
      ```


- Overview
- Package
- Class
- Use
- Tree
- Deprecated
- Index
- Help

- Prev Class
- Next Class

- Frames
- No Frames

- All Classes

- Summary:
- Nested |
- Field |
- Constr |
- Method

- Detail:
- Field |
- Constr |
- Method
